# Irinotecan plus carboplatin for patients with carcinoma of unknown primary site

**DOI:** 10.1038/sj.bjc.6604829

**Published:** 2008-12-16

**Authors:** K Yonemori, M Ando, M Yunokawa, T Hirata, T Kouno, C Shimizu, K Tamura, N Katsumata, A Hirakawa, K Matsumoto, Y Yamanaka, H Arioka, Y Fujiwara

**Affiliations:** 1 Breast and Medical Oncology Division, National Cancer Center Hospital, 5-1-1 Tsukiji, Chuo-ku, Tokyo 104-0045, Japan; 2 Department of Management Science, Graduate School of Engineering, Tokyo University of Science, 1-3 Kagurazaka, Shinjuku-ku, Tokyo 162-8601, Japan; 3 Medical Oncology Division, Hyogo Cancer Center, 13-70 Kitaouji-cho, Akasi, Hyogo 673-8558, Japan; 4 Medical Oncology Division, Tochigi Cancer Center, 4-9-13 Yonan, Utsunomiya, Tochigi 320-0834, Japan; 5 Medical Oncology Division, Yokohama Rosai Hospital, 3211 Kozukue, Kohoku-ku, Yokohama, Kanagawa 222-0036, Japan

**Keywords:** carboplatin, chemotherapy, irinotecan, unknown primary

## Abstract

Carcinoma of unknown primary site (CUP) is rarely encountered in clinical practice and optimal chemotherapy has not yet been established. This phase II study was conducted to evaluate the efficacy and toxicity of combined irinotecan+carboplatin therapy in chemotherapy-naive patients with CUP. Irinotecan was administered at 60 mg m^−2^ as a 90-min intravenous infusion on days 1, 8 and 15. Carboplatin was administered at an area-under-the curve of 5 mg ml^−1^ min as a 60-min intravenous infusion on day 1. This cycle was repeated every 28 days for up to six cycles. Forty-five patients were enrolled in the study. An intent-to-treat analysis revealed an objective response rate to the treatment of 41.9% (95% confidence interval, 27.0–57.9%). The median time to progression was 4.8 months and the median survival was 12.2 months. The 1- and 2-year survival rates were 44 and 27%, respectively. The most frequent grade 3 or more severe adverse events were leukopaenia (21%), neutropaenia (33%), anaemia (25%) and thrombocytopaenia (20%). Thus, the combination of irinotecan plus carboplatin was found to be active in patients with CUP. Therefore, the regimen may be one of the potentially available chemotherapeutic options for community standard of care in patients with a good performance status.

Carcinoma of unknown primary site (CUP) represents a group of heterogeneous malignancies that is diagnosed based on the presence of a metastatic disease without an identifiable primary tumour at the time of presentation. Carcinoma of unknown primary site accounts for approximately 3–5% of all newly diagnosed patients with malignancies (Briasoulis *et al*, 2008b).

The prognosis of CUP is generally poor, with a median overall survival time (OS) of approximately 6–12 months. Some of these patients with favourable and unique clinical and/or pathologic features may show prolonged survival with specific treatment approaches (Pavlidis *et al*, 2003). However, most of the patients fit into the category of poor prognosis. Many investigators have made efforts to develop optimal chemotherapeutic regimens based on the empiric approach, and platinum-based combination chemotherapy is considered to be one of the suitable treatment options for a large proportion of these patients (Pavlidis *et al*, 2003).

Irinotecan is a potent inhibitor of DNA topoisomerase I. It exhibits excellent antitumour activity, not only against a broad spectrum of tumours in experimental models (Kano *et al*, 1992; Misawa *et al*, 1995). Carboplatin is an analogue of cisplatin, with less severe non-haematological toxicities (Briasoulis *et al*, 2000; Yonemori *et al*, 2005). No cross-resistance has been found between irinotecan and carboplatin, and a synergistic effect of irinotecan with carboplatin has been shown in *in vitro* studies (Kano *et al*, 1993).

In an earlier study conducted by us, although the combination of docetaxel plus cisplatin produced favourable results in patients with CUP, treatment discontinuation sometimes became necessary because of the renal toxicity induced by cisplatin (Mukai *et al*, 2003; Yakushiji *et al*, 2006). Carboplatin has proven to be as effective as cisplatin against chemosensitive CUP, with an additional advantage of being better tolerated and more convenient in clinical practice (Briasoulis *et al*, 2000). In this study, we report the results of a phase II trial conducted to evaluate the effect of irinotecan plus carboplatin in the treatment for CUP.

## Patients and methods

### Patients

Patients who had histologically confirmed metastatic carcinoma were eligible for enrollment in this study, if the following evaluations did not reveal a primary site: complete history, physical examination, blood counts and blood chemistry examinations, including serum *α*-fetoprotein (AFP) and *β*-human chorionic gonadotropin (*β*-HCG) as tumour markers in both sexes, carbohydrate antigen 125 (CA125) as a tumour marker in women, prostate-specific antigen (PSA) as a tumour marker in men, urinalysis, head and neck examination with pharyngeal endoscopy conducted by experienced head and neck surgeons, urologic examination conducted by experienced urologists, mammography in women, gynaecologic examination by experienced gynaecologists in women, chest X-ray, whole-body computed tomography, upper gastrointestinal endoscopy, lower gastrointestinal endoscopy or barium enema, bone scintigraphy and direct workup of any symptomatic area.

Patients were enrolled in the study if they fulfilled the following eligibility criteria: (1) diagnosed as having CUP, (2) chemotherapy naive, (3) age⩾20 years, (4) life expectancy of at least 3 months, (5) an Eastern Cooperative Oncology Group performance status of ⩽2, (6) the presence of a measurable lesion as assessed by Response Evaluation Criteria in Solid Tumors (RECIST) (Therasse *et al*, 2000) and (7) adequate organ function (total leukocyte count⩾3000 per *μ*l or absolute neutrophil count⩾1500 per *μ*l, platelet count⩾100 000 per *μ*l, serum total bilirubin⩽1.5 mg dl^−1^, serum alanine aminotransferase⩽2 times the upper limit of normal, serum creatinine⩽1.5 mg dl^−1^). Patients with active infection, bowel obstruction, interstitial pneumonitis, uncontrolled severe heart disease, uncontrolled diabetes mellitus, pregnant or lactating women, symptomatic brain metastasis, severe coexistent medical illness or a past history of hypersensitivity to drugs were excluded from the study. Patients who had massive pleural effusion or ascites that required drainage or active concomitant malignancy were also excluded. Patient subgroups that were suitable for well-established treatments (i.e., men with blastic bone metastases showing features of adenocarcinoma and elevated PSA, women with axillary lymph nodes as the only site of disease showing features of adenocarcinoma, woman with papillary serous carcinoma of the peritoneum, patients with either cervical or inguinal lymph node involvement only with features of squamous cell carcinoma, patients with poorly differentiated carcinomas suggestive of germ cell tumour with elevated levels of AFP and/or *β*-HCG, patients with low-grade, well-differentiated neuroendocrine carcinoma and patients with carcinoma involving a single, potentially resectable site) were also excluded from the study. The protocol was approved by the institutional review board. All patients provided written informed consent before their enrollment.

### Treatment

Irinotecan was administered at the dose of 60 mg m^−2^ dissolved in 100 ml saline as a 90-min intravenous infusion, followed by carboplatin at an area-under-the curve of 5 mg ml^−1^ min dissolved in 250 ml of saline or 5% dextrose as a 60-min intravenous infusion. Irinotecan administration was planned for days 1, 8 and 15 of each cycle, and that of carboplatin was planned for day 1 of each cycle. The Calvert formula was used to determine the carboplatin dose, based on the glomerular filtration rate calculated using the serum creatinine level, body weight, age and sex (Cockcroft and Gault, 1976; Calvert *et al*, 1989). Patients showing treatment response or stable disease were administered up to a total of six courses. Granisetron 3 mg and dexamethasone 8 mg were used routinely before the drug infusions as antiemetic agents on days 1, 8 and 15. Prophylactic granulocyte colony-stimulating factor was not used routinely.

Irinotecan and carboplatin were administered on day 1 if the leukocyte count was ⩾3000 per *μ*l or the neutrophil count was ⩾1500 per *μ*l, the platelet count was ⩾75 000 per *μ*l, serum total bilirubin was ⩽1.5 mg dl^−1^, serum alanine aminotransferase was ⩽2 times the upper limit of normal, the serum creatinine was ⩽1.5 mg dl^−1^ and any non-haematological toxicities, with the exception of alopaecia, were ⩽grade 1. Patients who failed to improve to less than grade 2 in terms of the non-haematological toxicity even after withholding of the treatment for 2 weeks were withdrawn from the study.

Irinotecan was administered on day 8 or 15 if the leukocyte count was ⩾2000 per *μ*l or the neutrophil count was ⩾1000 per *μ*l, the platelet count was ⩾75 000 per *μ*l and any non-haematological toxicities, with the exception of alopaecia, were ⩽grade 1. The dose on day 8 and/or day 15 was omitted entirely if the counts or toxicities did not satisfy the above criteria.

Dose modification of carboplatin from AUC 4 to AUC 5 was allowed if febrile neutropaenia or grade 4 thrombocytopaenia was observed, or if platelet transfusion was required.

### Response and toxicity evaluation

All patients were re-evaluated for response after completion of two cycles of treatment, and the response categories were assigned based on the RECIST criteria (Therasse *et al*, 2000). Repeat scans at 8-week intervals were performed to confirm the response. The final response category assigned to these patients represented the best response obtained during the treatment course. Toxicities were evaluated according to the National Cancer Institute’s Common Toxicity Criteria, Version 2.0, after every cycle and at the end of the study treatment.

### Statistical analysis

The primary end point of this study was the objective response rate, defined as the proportion of patients with complete response or partial response in the intent-to-treat (ITT) population, in turn, defined as patients who had received at least one cycle of irinotecan and carboplatin. The secondary end points included safety and tolerability, time to tumour progression (TTP), OS, and the 1- and 2-year survival rates.

The sample size was determined using Simon’s Minimax two-stage design for phase II studies. The response rates to chemotherapy of patients with CUP have been reported as approximately in the range of 20–40% (Briasoulis *et al*, 2000; Greco *et al*, 2000a, 2000b; Dowell *et al*, 2001), so that the null hypothesis was that the true response rate was less than or equal to 30% (not considered to be clinically meaningful). The alternative hypothesis was that the true response rate was more than or equal to 50%. A total of 39 patients were required as the target sample to ensure results with 80% power and a type I error rate of 5%, for rejecting the null hypothesis that the true response probability was less than or equal to 30%. The enrollment of 45 patients was planned to fulfill the requirement of 39 patients, because some patients might need to be potentially excluded from the analysis because of failure to receive at least one cycle of irinotecan and carboplatin.

The objective response rate was reported as a percentage, along with the 95% confidence interval. The TTP and OS were determined by the Kaplan–Meier method. All the statistical analyses were performed using SPSS 12.0J (SPSS Inc., Chicago, IL, USA).

## Results

### Patient characteristics

Between May 2003 and November 2007, 45 patients were enrolled in this clinical trial. The patient characteristics are listed in Table 1. The median age was 59 years (range, 36–78 years), and the median performance status (PS) was 1 (range, 0–2). The median number of disease sites per patient was two (range, 1–7).

Twenty-three patients had lymph node involvement only. Serum tumour markers were assessed at the baseline pretreatment evaluation in 43 patients. The median number of tumour markers showing elevated serum levels was 5 (range, 0–10). Eighty-seven percent (*N*=39) of the patients showed elevated serum levels of tumour markers at the time of diagnosis (Table 2).

### Efficacy

**Figure 1:**
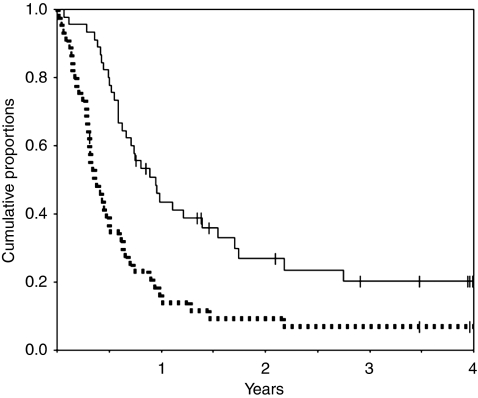
Kaplan–Meier analysis to determine the time to progression (dotted line) and overall survival (solid line).

Forty-five patients were enrolled in this study. All the enrolled patients were included in the analysis for TTP and OS, and 43 patients who had received at least one cycle of irinotecan plus carboplatin were assessed for tumour response to treatment. Two patients who were withdrawn from the study because of the appearance of toxicity in cycle 1 were considered as not evaluable. Objective response was observed in 18 patients, including complete response in two and partial response in 16 patients. Stable disease was observed in 10 patients and progressive disease in 15 patients. The results of an ITT analysis revealed an objective response rate of 41.9% (95% confidence interval, 27.0–57.9%); the response rate was 41.3% in the 30 patients with well-to-poorly differentiated adenocarcinoma and 50.0% in the 23 patients with lymph node involvement only. The median TTP was 4.8 months, and the median OS was 12.2 months. The 1- and 2-year survival rates were 44 and 27%, respectively (Figure 1).

### Toxicity

The toxicity data are listed in Table 3. Bone marrow suppression (leukopaenia, neutropaenia and thrombocytopaenia) and gastrointestinal toxicities, such as nausea, vomiting, diarrhoea and appetite loss, were the most frequent. There were no treatment-related deaths in this study.

Overall, 180 treatment cycles were administered and the median number of cycles per patient was four (range, 1–6). Of the 180 cycles, in 9.4% (17 episodes), the day-8 administration of irinotecan was withheld because of neutropaenia (11.8%), anaemia (5.9%), thrombocytopaenia (35.3%) or non-haematological toxicity (41.1%), including two episodes of fatigue, three episodes of nausea, two episodes of infection and one episode of palpitation. Furthermore, in 27.2% of the cycles, the day-15 administration of irinotecan was withheld because of neutropaenia (14.3%), thrombocytopaenia (65.3%), non-haematological toxicity (16.3%), including one episode of appetite loss, one episode of nausea, two episodes of diarrhoea, four episodes of febrile neutropaenia and patient refusal for personal reasons (two instances). The day-8 or day-15 irinotecan was withheld at least once in 24 (53%) patients. Five patients (11.1%) with anaemia required red blood cell transfusion and four patients (8.9%) with thrombocytopaenia required platelet transfusion. Dose modification of carboplatin was necessary in 15.5% of the patients (seven patients).

## Discussion

Recently published trials, in the literature, of regimens containing platinum agents for CUP have reported objective response rates in the range of 13–55% and median OS in the range of 6.0–16.2 months (Table 4). In two of these trials conducted to evaluate the activity of first-line platinum-based combination chemotherapy, the treatment regimen included irinotecan (Culine *et al*, 2003; Briasoulis *et al*, 2008a). According to one, the combination of irinotecan plus cisplatin yielded an objective response rate of 38% and median OS of 6 months (Culine *et al*, 2003). In another study limited to poor-prognosis patients, irinotecan plus oxaliplatin yielded an objective response rate of 13% and median survival time of 9.5 months, with 40% of the patients still alive at 1 year (Briasoulis *et al*, 2008a). The patient background, especially the prognostic characteristics, may have an influence on the treatment outcome. Two-thirds of the patients in this study were prognostically good-risk patients, with a low percentage of patients having liver metastasis and a large percentage of patients with the disease extent being limited to the lymph nodes; in contrast, in most of the recently published series, the majority of the patients were prognostically poor-risk patients and/or had liver metastasis. Therefore, potential bias would make a reliable comparison of the results of the present and previous studies difficult.

Interestingly, the Kaplan–Meier analysis in this study revealed a 2-year survival rate of 27%, with some patients even showing long-term survival (Figure 1). The results of chemotherapy in a total of 1515 patients enrolled in 45 trials including 10 patients or more conducted between 1964 and 2002 showed that survival of the patients beyond 2 years was rare and that there were no cases of disease-free survival beyond 3 years (Pavlidis *et al*, 2003; Greco and Hainsworth, 2008). However, more recent studies have reported long-term survival in a small percentage of patients (Table 4). Long-term follow-up of the 396 patients enrolled in the five most recent studies revealed 1-, 2-, 3-, 5-, 8- and 10-year survival rates of 38, 19, 12, 11, 8 and 8% (Greco and Hainsworth, 2008). Although the reasons for the recent increase in long-term survival are uncertain, it is noteworthy that long-term survival was obtained with the combination of platinum agents and new agents.

The emergence of new non-platinum agents after 1995, including taxanes, gemcitabine, vinorelbine and irinotecan, has enabled the development of platinum-based combination chemotherapy for patients with CUP (Pavlidis *et al*, 2003). However, no definitive conclusions have been reached, because there is still no evidence based on randomised clinical trials to prove the superiority of the aforementioned combination chemotherapies over single-agent platinum therapy. In addition, the clinical benefits and risks of doublet and triplet combination chemotherapies are still uncertain. An attempt was made by European investigators to compare the effect of single-agent cisplatin with that of combined therapy with gemcitabine plus cisplatin on survival in good-risk patients with CUP. Although the results of this prospective trial were expected to clarify the role of combination chemotherapy in good-risk patients with CUP, the trial was stopped due to insufficient accrual, and the result showed a non-significantly higher survival with gemcitabine plus cisplatin as compared to that with cisplatin alone (Gross-Goupil *et al*, 2008).

Recently, standard chemotherapeutic regimens with or without molecular-targeting agents have been established for many cancers. Thus, there is a great demand to optimise the chemotherapeutic regimen for each patient with CUP. The approach based on the genomic characteristics may come to represent one of the breakthroughs in the proper use of chemotherapies tailored to individual patients.

In addition, the advances in the development of many molecular-targeted agents provide opportunities to explore various new combination therapies containing both cytotoxic and molecular-targeted agents for patients with CUP. Several studies have demonstrated the immunohistochemical expression of relevant molecular targets at high frequencies in tissue specimens (Massard *et al*, 2007). A phase II trial of bevacizumab plus erlotinib revealed substantial activity of this combination in patients treated previously or patients who had not received treatment because of the presence of poor-prognostic features (Hainsworth *et al*, 2007). In a preliminary study, treatment with paclitaxel plus carboplatin used in combination with bevacizumab plus erlotinib yielded an objective response rate of 48% (*N*=19 out of 40) and was well tolerated as first-line chemotherapy for patients with CUP (Greco *et al*, 2008). After first-line platinum-based combination chemotherapy, the approach of empiric second-line chemotherapy has shown little promise, with extremely low response rates (Hainsworth *et al*, 2001, 2005). Therefore, tailor-made first-line chemotherapy by genomic typing or addition of molecular-targeted drugs may be important in the treatment of CUP, which includes heterogeneous cancers, rather than the development of second-line chemotherapy.

In this study, the most frequently encountered toxicity was haematological toxicity and some patients needed blood transfusion or dose reduction of carboplatin. The dose delivery was fairly smooth in the chemotherapy-naive patients with CUP as compared with that in our earlier phase I study of combined irinotecan plus carboplatin in patients with heavily treated ovarian cancer (Yonemori *et al*, 2005). Among the advantages of this regimen are that it is easy to adjust the irinotecan dose during each chemotherapy cycle according to the individual toxicity profiles and to manage the chemotherapy on an outpatient basis without prophylactic use of granulocyte-stimulating factor or erythropoietin.

In conclusion, combined irinotecan plus carboplatin chemotherapy appears to exert satisfactory activity and to be reasonably well tolerated in patients with CUP. Many conventional chemotherapies have been reported as the community standard of care for patients with CUP. This regimen was moderately well tolerated and may become established as one of the treatment options in patients with a good PS.

## Conflict of interest

Katsumata N: Honoraria (Sanofi-aventis, Pfizer, Nippon Kayaku, Kyowa Hakko Kogyo, Zeneca). His family members have no financial interest or conflict of interest in relation to this study. None of the authors or their immediate family members have any financial interest or conflict of interest in relation to this study.

## Figures and Tables

**Table 1:** Patient characteristics

**Characteristics**	**No. of patients**
No. of patients enrolled	45
	
*Age (years)*	
Median	59
Range	36–78
	
*Sex*	
Male	23
Female	22
	
*ECOG performance status*	
0	19
1	22
2	4
	
*Histologic type*	
Adenocarcinoma (well and moderately differentiated)	21
Poorly differentiated adenocarcinoma	9
Squamous cell carcinoma	7
Poorly differentiated carcinoma	5
Clear cell carcinoma	1
Small cell carcinoma	1
Undifferentiated carcinoma	1
	
*No. of disease sites*	
1	13
2	10
⩾3	22
	
*Site of disease*	
Lymph node	40
Lung	6
Bone	4
Liver	8
Adrenal	2
Malignant effusion	4
Soft tissue	3
Other	6
	
*Prognostic index*	
Culine *et al* (2002a) a	
Good risk	29
Poor risk	16
	
van der Gaast *et al* (1996) b	
Good risk	19
Intermediate risk	19
Poor risk	7

ECOG=Eastern Cooperative Oncology Group.

a Good-risk patients had a performance status of 0 or 1 and normal serum lactate dehydrogenase (LDH) levels; poor-risk patients had a performance status of ⩾2 or elevated serum LDH levels.

b Good-risk patients had a performance status of 0 and serum alkaline phosphatase (ALP) levels of <1.25 × normal range (*N*); intermediate-risk patients had a performance status of ⩾1 or serum ALP levels of ⩾1.25 × *N*; poor-risk patients had a performance status of ⩾1 and serum ALP levels of ⩾1.25 × *N*.

**Table 2:** Elevated serum tumour marker levels at diagnosis

**Markers**	**Normal range**	**No. of measured patients**	**No. of patients with elevated levels (%)**
AFP	⩽10 ng ml^−1^	42	2 (4.7)
*β*-HCG	⩽0.5 mIU ml^−1^	42	22 (52.4)
Cyfra	⩽2.2 ng ml^−1^	41	30 (73.2)
SCC	⩽1.5 ng ml^−1^	41	7 (17.1)
NSE	⩽15 ng ml^−1^	42	10 (23.8)
ProGRP	<46 pg ml^−1^	41	8 (19.5)
PSA	⩽2.7 ng ml^−1^	23	5 (21.7)
CEA	⩽5.0 ng ml^−1^	43	19 (44.2)
SLX	⩽38 U ml^−1^	41	21 (51.2)
STN	⩽45 U ml^−1^	41	16 (39)
NCC-ST439	⩽4.5 U ml^−1^	41	16 (39)
CA125	⩽35 U ml^−1^	39	25 (64.1)
CA15-3	⩽28 U ml^−1^	41	12 (29.3)
CA19-9	⩽37 U ml^−1^	43	17 (39.5)
PIVKA-II	<40 mIU ml^−1^	39	2 (5.1)
Elastase	⩽300 ng dl^−1^	41	3 (7.3)

AFP=*α*-fetoprotein; CA125=carbohydrate antigen 125; CA15-3=carbohydrate antigen 15-3; CA19-9=carbohydrate antigen 19-9; CEA=carcinoembryonic antigen; Cyfra=cytokeratin 19 fragment; NCC-ST439=national cancer center-ST439; NSE=neuron-specific antigen; PIVKA-II=protein induced by vitamin K absence-2; ProGRP=progastrin-releasing peptide; PSA=prostate-specific antigen; SCC=squamous-cell carcinoma antigen; SLX=sialyl-specific embryonic antigen; STN=sialyl TN antigen; *β*-HCG=*β*-human chorionic gonadotropin.

**Table 3:** Toxicity profiles (frequency>10%)

**Profile**	**Frequency (%)**	**No. of grade 3 (%)**	**No. of grade 4 (%)**
*Haematologic toxicity*			
Leukopaenia	75.6	6 (13.3)	4 (8)
Neutropaenia	80	6 (13.3)	9 (20)
Anaemia	93.3	8 (17.8)	3 (6.7)
Thrombocytopaenia	68.9	7 (15.6)	2 (4.4)
			
*Non-haematologic toxicity*			
Fatigue	60	0 (0)	0 (0)
Appetite loss	46.7	0 (0)	0 (0)
Nausea	82.2	1 (2.2)	0 (0)
Vomiting	26.7	1 (2.2)	0 (0)
Diarrhoea	57.8	4 (8)	0 (0)
Constipation	42.2	0 (0)	0 (0)
Skin rash	20	0 (0)	0 (0)
Febrile neutropaenia	13.3	5 (11.1)	1 (2.2)

**Table 4:** Clinical trials of first-line regimens containing platinum agents reported in the literature from 2000

**Group**	**Reference**	**Regimen**	***N***	**RR**	**MST (m)**	**1 year^a^**	**2 year^b^**
Doublet	Briasoulis *et al* (2000)	Carbo–P	77	38.7%	13.0	NA	NA
	Voog *et al* (2000)	Cis–E	22	32%	8.0	NA	NA
	Greco *et al* (2000a)	Cis–D	26	26%	8.0	40%	28%
		Carbo–D	47	22%	8.0	33%	28%
	Dowell *et al* (2001)	Carbo–E	17	19%	8.3	26%	NA
	Saghatchian *et al* (2001)	Cis–E → Cis–E–B–I	30	40%	9.4	NA	28%
		Cis–F	18	44%	16.1	NA	39%
	Culine *et al* (2002b)	Dx–Cy⇔Cis–E	82	39%	10.0	NA	NA
	Culine *et al* (2003)	Cis–G	39	55%	8.0	NA	NA
		Cis–Ir	40	38%	6.0	NA	NA
	Park *et al* (2004)	Cis–P	37	42%	11.0	38%	11%
	El-Rayes *et al* (2005)	Carbo–P	22	23%	6.5	27%	NA
	Pittman *et al* (2006)	Carbo–G	51	30.5%	7.8	26%	12%
	Briasoulis *et al* (2008a)	Ox–Ir	47	13%	9.5	40%	NA
	Pentheroudakis *et al* (2008)	Carbo–D	47	32%	16.2	NA	NA
	This study	Carbo–Ir	45	41.9%	12.2	44%	27%
Triplet or more	Parnis *et al* (2000)	Cis–F–Ep	43	23%	5.8	NA	NA
	Greco *et al* (2000b)	Carb–P–E	71	48%	11.0	48%	20%
	Guardiola *et al* (2001)	Cis–Dx–Cy	22	50%	10.7	NA	NA
	Macdonald *et al* (2002)	Cis–F–Mit	31	27%	7.7	28%	10%
	Greco *et al* (2002)	Carbo–G–P	113	25%	9.0	42%	23%
	Balaña *et al* (2003)	Cis–G–E	30	36.6%	7.2	26%	NA
	Piga *et al* (2004)	Carbo–Dx–E	102	26.5%	9.0	35.2%	18.1%
	Greco *et al* (2004)	Carbo–P–E → G–Ir	111	33%	9.1	35%	16%
	Palmeri *et al* (2006)	Cis–G–P	33	48.5%	9.6	NA	NA
		Cis–G–V	33	42.3%	13.6	NA	NA
	Schneider *et al* (2007)	Carb–G–Cape	33	39.4%	7.6	35.6%	14.2%
	Greco *et al* (2008)	Carbo–P–Bv–Er	51	48%	11.3	NA	NA

B=bleomycin; Bv=bevacizumab; Cape=capecitabine; Carbo=carboplatin; Cis=cisplatin; Cy=cyclophosphamide; D=docetaxel; Dx=doxorubicin; E=etoposide; Ep=epirubicin; Er=erlotinib; F=5-FU; G=gemcitabine; I=ifosfamide; Ir=irinotecan; m=months; Mit=mitomycin C; MST=median survival time; NA=not available; Ox=oxaliplatin; P=paclitaxel; RR=response rate; V=vinorelbine.

a 1 year=1-year survival rate.

b 2 year=2-year survival rate.
